# Preparation, Characterization, and Mechanism of Hypoglycemic Action of a Goat Casein Peptide Delivery System Involving DPP-IV Inhibition and GLP-1 Release

**DOI:** 10.3390/foods14213795

**Published:** 2025-11-05

**Authors:** Xiaojing Du, Wenlin Niu, Hongxin Wang

**Affiliations:** 1 National Engineering Research Center of Wheat and Corn Further Processing, Henan University of Technology, Zhengzhou 450001, China; 2 College of Food Science and Engineering, Henan University of Technology, Zhengzhou 450001, China; 3 The State Key Laboratory of Food Science & Technology, Jiangnan University, Wuxi 214122, China; 4 Collaborative Innovation Center of Food Safety and Quality Control in Jiangsu Province, Jiangnan University, Wuxi 214122, China

**Keywords:** goat casein peptides, liposomes, niosomes, bioaccessibility, gut microbiota

## Abstract

This study aimed to formulate a carrier system to improve the oral bioaccessibility of goat casein peptides (GCAPS). Goat casein was hydrolyzed with papain and subsequently purified to obtain bioactive peptide fractions (GCAPS) with potent hypoglycemic activity. On this basis, spherical GCAPS-loaded nanocarrier systems were developed, including liposomes (GCAPS-LS) and niosomes (GCAPS-NS). Among them, GCAPS-NS exhibited higher encapsulation efficiency (94.98 ± 3.01%) and a smaller particle size (89.81 ± 8.97 nm) than GCAPS-LS. FT-IR analysis confirmed successful peptide encapsulation. Simulated gastrointestinal digestion experiments demonstrated that GCAPS-NS significantly improved GCAPS retention and DPP-IV inhibition. In vivo results from high-fat diet-induced (HFD) insulin-resistant mice demonstrated that GCAPS-NS effectively ameliorated metabolic abnormalities by including adiposity, enhancing GLP-1 levels and suppressing hsCRP expression, thereby contributing to improved glycemic homeostasis. Moreover, GCAPS-NS intervention resulted in a significant enrichment of *Akkermansia* and a reduced *Firmicutes*/*Bacteroidetes* ratio, suggesting its beneficial role in alleviating HFD gut dysbiosis. These findings indicated that goat casein peptides held great potential as a functional food for the management of type 2 diabetes.

## 1. Introduction

Type 2 diabetes mellitus (T2DM) is a complex chronic metabolic disorder characterized primarily by impaired carbohydrate metabolism. As the most common form of diabetes, T2DM accounts for approximately 90% of all cases and is closely associated with obesity [1]. Currently, oral hypoglycemic agents remain the mainstay of T2DM treatment. Despite their efficacy in controlling blood glucose levels, their use is often accompanied by adverse effects, including allergic reactions and gastrointestinal disturbances. In recent years, incretin-based therapies, particularly GLP-1 receptor agonists and DPP-IV inhibitors, have emerged as a focal point in diabetes treatment research owing to their capacity to prolong endogenous GLP-1 activity, effectively control postprandial glucose levels, and demonstrate favorable safety and tolerability profiles [2]. However, the high cost of synthetic DPP-IV inhibitors and concerns regarding their long-term safety remain significant issues. In contrast, DPP-IV inhibitors derived from natural food sources are generally considered to have superior safety profiles and minimal side effects, presenting great potential for the prevention and adjunctive management of type 2 diabetes mellitus and other chronic metabolic disorders. Therefore, identifying safe, food-derived bioactive compounds with antidiabetic potential has emerged as a major focus of current research.

Bioactive peptides exert significant physiological functions related to metabolic regulation and human health maintenance [1]. However, their instability under processing and digestive conditions poses a significant barrier to their practical use in food systems [3]. Therefore, the development of delivery systems capable of significantly enhancing the long-term stability of bioactive peptides is of great importance for expanding their potential in practical applications. Encapsulation of peptides within delivery systems not only enhances their stability and bioaccessibility but also effectively masks undesirable bitterness [4,5]. Nanocarriers can enhance epithelial permeability and traverse the blood–brain barrier through receptor-mediated transcytosis. This process involves binding to specific receptors, such as transferrin or insulin receptors, which facilitates endocytosis and transcellular transport, thereby enabling efficient and targeted systemic delivery rather than relying solely on nonspecific paracellular diffusion [6]. Compared to microcapsules, nanocapsules enable enhanced gastrointestinal absorption, sustained release, and improved site-specific delivery of bioactive compounds [7]. Accordingly, various nanocarriers have been investigated as promising delivery systems for proteins and peptides, including solid lipid nanoparticles, pickering double emulsions, and liposomes [8,9,10,11]. Among these nanocarriers, liposomes are regarded as particularly suitable for peptide encapsulation owing to their amphiphilic nature and excellent biocompatibility [12,13]. However, conventional liposomes face several limitations, including aggregation, fusion, and precipitation, which compromise their scalability in food-based systems [14]. Niosomes are bilayer vesicles composed of non-ionic surfactants and cholesterol, and they functionally and structurally resemble liposomes [14]. Niosomes retain the amphiphilic properties and encapsulation capacity of liposomes, while providing enhanced stability and biocompatibility that help mitigate the toxicity arising from phospholipid oxidation [15].

In this study, GCAPS-LS and GCAPS-NS were developed as protective delivery systems for goat casein-derived peptides (GCAPS), aiming to enhance their stability and bioaccessibility. Accordingly, the in vivo DPP-IV inhibition and GLP-1 secretion levels of GCAPS and GCAPS-NS were further evaluated in mice, with particular emphasis on their regulatory roles in glucose metabolism and gut microbiota modulation. This study offers a theoretical basis and practical guidance for the development and application of goat milk-derived bioactive peptides in metabolic health management.

## 2. Materials and Methods

### 2.1. Materials and Methods

Goat milk flour was supplied by Guangzhou Xingfu Yizhan Brand Management Co., Ltd. (Guangzhou, China), and all materials used in this study were obtained from the same batch. Consistent procedures were applied during casein isolation and hydrolysis to ensure the stability and reliability of the subsequent bioactivity results. Rennet (890 IMCU/mL) was purchased from SACCO System. Commercial kits for triglycerides (TG), total cholesterol (TC), High-density lipoprotein cholesterol (HDL-C), and low-density lipoprotein cholesterol (LDL-C) were sourced from Jiangsu Edison Biotechnology Co., Ltd. (Nanjing, China). Trizol reagent, hsCRP ELISA kits, and TNF-α ELISA kits (mouse) were obtained from Beyotime Biotechnology (Shanghai, China). A high-fat diet (60% energy from fat) was provided by Jiangsu Synergy Pharmaceutical Bioengineering Co., Ltd. (Nanjing, China).

### 2.2. Preparation of Goat Casein Peptide (GCAPS)

Goat casein-derived peptides (GCAPS) were obtained as reported in our previous study [1]. Briefly, goat milk powder was dispersed in ultrapure water and centrifuged at 6000 rpm for 15 min to obtain defatted goat milk. Rennet was added to the defatted goat milk at a final concentration of 0.8% (*w*/*v*), and the mixture was incubated for 15 min to induce casein coagulation.

The casein was prepared into a dispersion with a mass concentration of 30 mg/mL, and papain was added to make the final enzyme substrate ratio of 9000 U/g (E/S) for enzymatic hydrolysis. Papain was performed at 55 °C and pH 7.0 for 4 h. The pH was maintained constant by titrating with 0.1 mol/L NaOH or HCl during the reaction. After hydrolysis, the enzyme was inactivated in a boiling water bath for 10 min, and the mixture was centrifuged at 8000 rpm for 30 min. The resulting supernatant was subsequently fractionated using a 3 kDa molecular weight cut-off ultrafiltration membrane (Sigma-Aldrich, Shanghai, China).

The sample was further purified by Sephadex gel filtration chromatography and RP-HPLC to obtain purified goat casein peptides, designated as GCAPS. The specific experimental parameters were documented in the Supplementary Materials.

### 2.3. Determination of Encapsulation EFFICIENCY (EE)

The encapsulation efficiency (EE) of GCAPS in the delivery systems was measured using the dialysis bag method [16]. The nanoencapsulated samples were dialyzed using 12 kDa MWCO membranes, and 1 mL aliquots were collected from inside the dialysis bag at 0.5 h intervals. The formula for calculating the EE is shown in the formula:

(1) $$\mathrm{EE}\;(\%)\;=\;[m_{1}\;-\;\frac{m_{2}}{m_{1}}]\;\times \;100$$

where *m*_1_ is the total amount of peptide, and *m*_2_ is the amount of free peptide.

### 2.4. Characterization of GCAPS Encapsulation System

#### 2.4.1. Characterization of Surface Morphology

The surface microstructure of the samples was characterized using field emission scanning electron microscopy (FE-SEM, SU8100, Hitachi High-Tech Co., Ltd., Xiasong, Japan), transmission electron microscopy (TEM, HT7700, Hitachi, Japan), and atomic force microscopy (AFM, Cypher ES, Asylum Research, Santa Barbara, CA, USA).

#### 2.4.2. Fourier Transform Infrared Spectroscopy (FT-IR)

Fourier transform infrared (FT-IR) spectroscopy (Antaris II, Thermo Fisher Scientific, Waltham, MA, USA) was employed to measure the samples. The specific experimental parameters were applied according to the previously reported method [17].

### 2.5. In Vitro Digestive Stability of the Nanoparticle System

A dialysis-based approach was employed to evaluate GCAPS retention within the nanoparticle system under simulated gastrointestinal conditions. Simulated gastric fluid (SGF) and intestinal fluid (SIF) were prepared [18]. A 3 mL sample sealed in a dialysis bag was sequentially incubated in SGF (pH 1.2, 37 °C) and then in SIF (PBS, pH 7.4) for 2 h each at 120 rpm. During digestion, 0.5 mL of dialysate was collected every 30 min and replaced with an equal volume of fresh sample. Enzymatic activity was terminated by heating the samples in a boiling water bath for 5 min, followed by centrifugation at 5000 rpm for 20 min. The GCAPS retention rate and bioaccessibility were calculated using the formula:

(2) $$\mathrm{GCAPS}\;\mathrm{retention}\;\mathrm{rate}\;(\%)\;=\;\frac{A_{0}}{A_{1}}\;\times \;100\%$$

(3) $$\mathrm{Bioaccessibility}\;\mathrm{of}\;\mathrm{GCAPS}\;(\%)=\frac{A_{1}}{A_{2}}\;\times \;100\%$$

where *A*_0_ is the peptide content before digestion, *A*_1_ is the GCAPS in the dispersion after digestion, and *A*_2_ is the initial total GCAPS input.

### 2.6. Determination of DPP-IV Inhibition

The determination of DPP-IV inhibition was carried out based on our previously reported method [17]. The sample or the positive control, isoleucine–proline–isoleucine (IPI), was prepared as a 2 mg/mL solution. An aliquot of 100 μL of the sample solution was mixed with 1.5 mmol/L Gly-Pro-p-nitroanilide and incubated at 37 °C for 12 min. Subsequently, 150 μL of DPP-IV enzyme solution (8 U/mL) was added, and the reaction was continued for 60 min. The enzyme was then inactivated by placing the reaction mixture in a boiling water bath for 5 min. The absorbance at 405 nm was measured for the sample group (*S*), sample blank (*S*_0_), control group (*C*), and control blank (*C*_0_). The DPP-IV inhibitory activity was calculated according to the following equation.

(4) $$\mathrm{DPP-IV}\;\mathrm{inhibition}(\%)=[1-(\frac{S-S_{0}}{C-C_{0}})]\;\times \;100$$

### 2.7. In Vivo Bioavailability of GCAPS and GCAPS-NS

#### 2.7.1. Animal Experiment

72 SPF-grade male C57BL/6J mice (6 weeks old, 20–25 g) were employed in this study. The experimental protocol involving animals was reviewed and approved by the Animal Ethics Committee of Jiangnan University. The Animal Experimental Center of Jiangnan University is accredited by the Jiangsu Provincial Department of Science and Technology for the use of SPF-grade rodents (License No. SYXK (Su) 2021-0056), and was authorized to house SPF-grade C57BL/6J mice. The animal experiment approval number is JN.No20220315c0900525[011]. Animals were maintained under controlled conditions at 20–26 °C and 40–70% humidity for the duration of the experiment.

#### 2.7.2. Establishment of Animal Model and Drug Intervention

Mice with fasting blood glucose levels ranging from 10 to 25 mmol/L were considered successfully established hyperglycemic models. Following the successful establishment of insulin resistance through high-fat diet feeding, hyperglycemic mice were randomized into groups such that intergroup fasting blood glucose differences did not exceed 1.1 mmol/L. To ensure uniform distribution, mice were weight-matched and randomly assigned to 9 groups (*n* = 8 per group). One group was designated as the normal chow diet (NCD) control and received distilled water via oral gavage. Another group was maintained on an HFD and administered distilled water in the same manner. The remaining seven groups were fed an HFD and subjected to different oral interventions. One group was designated as the positive control (HFD+PC) and administered metformin at a dose of 625 mg/kg body weight (625 mg/kg body weight). Three groups were treated with GCAPS at 25, 50, and 150 mg/kg body weight, corresponding to low, medium, and high doses, designated as HFD+PSL, HFD+PSM, and HFD+PSH, respectively. Another three groups were administered GCAPS-NS at equivalent peptide doses and were designated as HFD+NSL, HFD+NSM, and HFD+NSH. All administered doses were within toxicologically safe limits.

#### 2.7.3. Sample Collection from Experimental Animals

Upon completion of the experiment, mice were fasted for 12 h following the final oral gavage, then anesthetized with inhalation of isoflurane (induction concentration 3–4%, maintenance concentration 1–1.5%) and humanely euthanized [19]. Blood samples were obtained by retro-orbital bleeding and centrifuged to isolate the serum. The collected supernatants were divided into sterile tubes and frozen at −80 °C for downstream applications. Blood was collected, and liver and epididymal adipose tissues were dissected and fixed in 4% paraformaldehyde solution. The calculations of the adiposity index and liver index were performed according to the formula:

(5) $$\mathrm{Adiposity}\;\mathrm{index}\;(\mathrm{mg}/g)\;=\;\frac{m_{1}}{m_{2}}$$

(6) $$\mathrm{Liver}\;\mathrm{index}\;(\mathrm{mg}/g)=\frac{m_{3}}{{\;m}_{2}}\;\times \;100$$

where *m*_1_ is the weight of adipose tissue, mg; *m*_2_ is the body weight of the mouse, g; and *m*_3_ is the liver weight of the mouse, mg.

#### 2.7.4. Determination of Serum and Hepatic Lipid Biochemical Parameters

Approximately 60 mg of liver tissue was precisely measured and transferred into a sterile microcentrifuge tube containing 1 mL of physiological saline and an appropriate amount of homogenization beads. The sample was vortexed for 30 s and subsequently centrifuged at 2000 rpm for 10 min to remove insoluble particulates. The supernatant was collected for the determination of TC and TG levels [19].

Serum samples were equilibrated to room temperature before HDL-C and LDL-C levels were measured using commercial kits according to the manufacturer’s instructions.

#### 2.7.5. Hematoxylin and Eosin Staining

The experimental protocol was conducted according to a previously reported method [20]. The liver and epididymal adipose tissues of mice were fixed in paraformaldehyde for 24 h, trimmed, and rinsed with tap water for 30 min. The tissues were dehydrated through a graded ethanol series and cleared in xylene. Cleared liver and pancreas samples were embedded in molten paraffin and allowed to solidify into blocks, which were then sectioned at a thickness of 4 μm using a microtome. Sections were floated on 45 °C water, mounted on glass slides, and dried at 65 °C. For staining, sections were deparaffinized with xylene and rehydrated through sequential immersion in absolute ethanol, 95% ethanol, and 80% ethanol for 10, 5, and 5 min, respectively, followed by rinsing under running water for 1 min. Hematoxylin staining was performed for 5 min, differentiated in 1% hydrochloric acid followed by ammonia water, and washed under running water for 1 h. Sections were subsequently dehydrated in 70% and 90% ethanol for 10 min each, counterstained with eosin for 3–5 min, dehydrated in absolute ethanol, cleared in xylene, and mounted with neutral resin. Coverslips were applied, and the sections were air-dried prior to microscopic observation.

#### 2.7.6. Oral Glucose Tolerance Test (GTT)

Mice were fasted for 12 h, after which baseline (0 h) blood glucose levels were measured. Glucose was then orally administered at a dose of 2.0 g/kg body weight within 20 min. Blood glucose levels were measured at regular intervals between 0.5, 1.0, 1.5, and 2.0 h after glucose administration. The changes in blood glucose and the area under the curve (AUC) were analyzed and compared between the model and treatment groups [21]. The AUC was calculated from the blood glucose time curve.

#### 2.7.7. Insulin Tolerance Test (ITT)

Mice were fasted for 6 h, after which baseline (0 h) blood glucose levels were recorded and insulin (0.75 U/kg body weight) was administered intraperitoneally. Blood glucose levels were measured at 0.5, 1, 1.5, and 2 h after injection [22]. Glucose levels and AUC were compared between the model and experimental groups.

#### 2.7.8. Determination of Fasting Blood Glucose Levels

At the end of the experiment, animals were fasted for 5 h before the final blood glucose measurement, followed by comparison of glucose levels and percentage reductions between groups [23]. The percentage reduction was calculated using the formula:

(7) $$\mathrm{Percentage}\;\mathrm{Reduction}\;\mathrm{in}\;\mathrm{Blood}\;\mathrm{Glucose}\;(\%)=\frac{{\;T}_{1}-T_{2}}{T_{1}}\;\times \;100\%$$

where *T*_1_ is the value of the initial glucose level, and *T*_2_ is the value of the final glucose level.

#### 2.7.9. Determination of Serum GLP-1 and hsCRP Levels

Following anesthesia and euthanasia, cardiac blood was collected from the mice and centrifuged at 2000 rpm for 10 min to obtain serum. GLP-1 (active) and hsCRP levels in serum were determined using commercial ELISA kits according to the manufacturer’s instructions.

### 2.8. Analysis of Gut Microbiota in Mice

Gut microbiota analysis for all sample groups was outsourced to Novogene Co., Ltd. (Beijing, China). The specific experimental conditions were documented in the Supplementary Materials.

### 2.9. Statistical Analysis

All experiments were performed in triplicate, and data are expressed as mean ± standard deviation (SD). Statistical comparisons among groups were conducted using one-way analysis of variance (one-way ANOVA) in SPSS 29.0 software (IBM Corp., Armonk, NY, USA). For measurements at different time points within the same group, data were first tested for normality (Shapiro–Wilk test) and sphericity (Mauchly’s test) before performing repeated-measures ANOVA. When the assumptions of sphericity were violated, appropriate corrections were applied to ensure the reliability of the results. Statistical significance was set at *p* < 0.05.

## 3. Results and Discussion

### 3.1. Preparation of GCAPS-Loaded Nanoparticle Systems (GCAPS-LS and GCAPS-NS)

The particle size, PDI, ζ-potential, and EE of various phytosterol-based formulations were compared. In the GCAPS-L system, cholesterol, phytosterol, ergosterol, β-sitosterol, and stigmasterol formulations were designated as GCAPS-LC, GCAPS-LP, GCAPS-LE, GCAPS-LS, and GCAPS-LG, respectively. In the GCAPS-N system, they were denoted as GCAPS-NC, GCAPS-NP, GCAPS-NE, GCAPS-NS, and GCAPS-NG. As shown in Figure S1, the liposome appeared milky and turbid, whereas the niosome exhibited a white and transparent appearance. Moreover, GCAPS-LS displayed a smaller particle size and enhanced size uniformity relative to the other samples. The ζ-potential measurements revealed that all formulations possessed absolute values greater than 25 mV, indicating favorable colloidal stability [17,24]. Moreover, GCAPS-LS and GCAPS-LC demonstrated higher EE than the other samples. Compared with the liposome, all niosome samples exhibited significantly smaller particle sizes. The average particle sizes of GCAPS-NC, GCAPS-NP, GCAPS-NE, GCAPS-NS, and GCAPS-NG were 138.6 ± 6.87, 92.5 ± 6.87, 102.9 ± 9.87, 89.8 ± 8.97, and 136.4 ± 8.76 nm, respectively. The corresponding encapsulation efficiencies were 83.22 ± 1.45%, 84.24 ± 3.21%, 87.67 ± 2.01%, 94.98 ± 3.01%, and 92.35 ± 2.35% (Figure S1). Niosome samples exhibited lower PDI values compared to the liposome, indicating better dispersibility in aqueous environments. In addition, the niosomes showed higher EE and smaller particle sizes than the liposomes, with GCAPS-NS demonstrating the most favorable characteristics. The effect of further optimization of β-sitosterol content on the EE and particle size of GCAPS was analyzed, and the optimization results are presented in Figure S2.

### 3.2. Characterization of the GCAPS-Loaded Nanoparticle System

**Figure 1:**
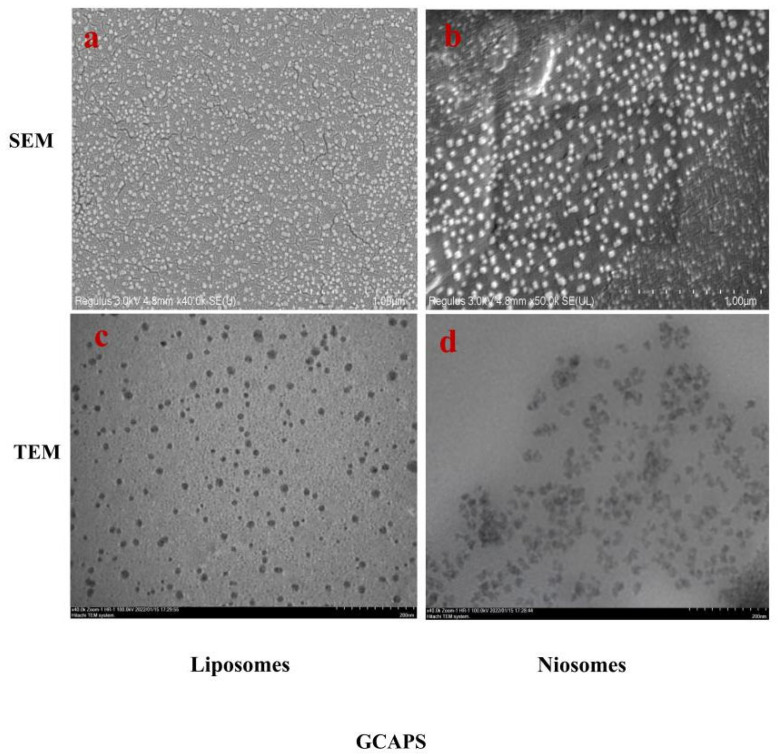
SEM (**a**,**b**) and TEM (**c**,**d**) images of GCAPS-LS and GCAPS-NS.

As shown in Figure 1a,b, both GCAPS-LS and GCAPS-NS exhibited smooth surfaces with uniform nanospherical morphology. Further morphological characterization by TEM (Figure 1c,d) revealed well-dispersed spherical particles for both encapsulation systems, indicating favorable structural integrity and dispersion stability.

**Figure 2:**
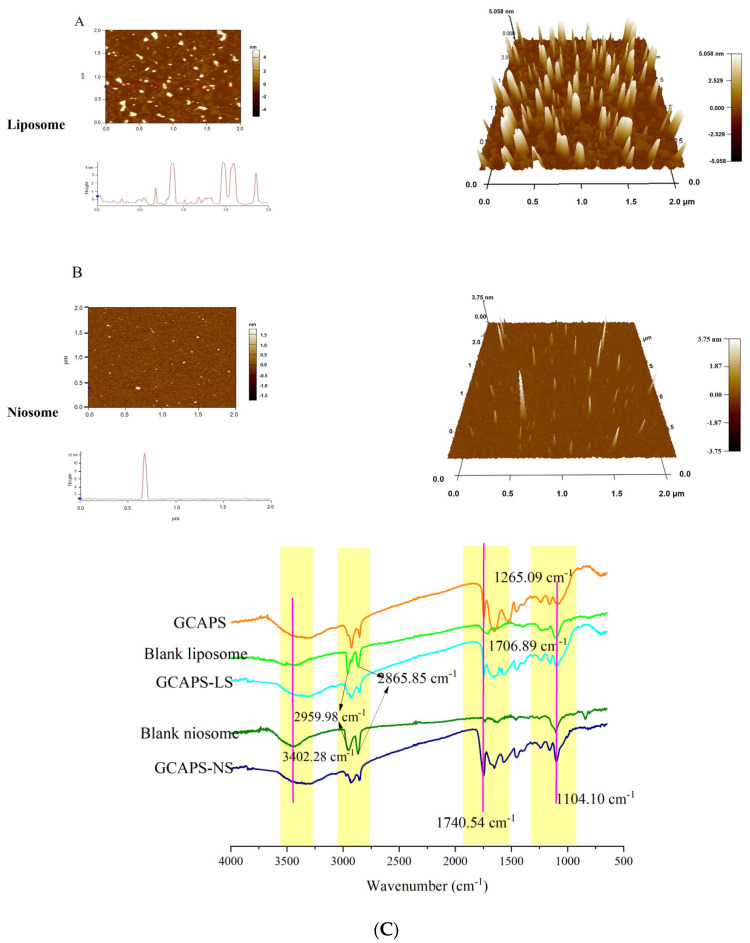
AFM images of GCAPS-LS and GCAPS-NS (**A**,**B**); The red line represents the height profile. FT-IR spectra of GCAPS and encapsulated systems (**C**).

As shown in Figure 2A,B, GCAPS-NS exhibited a compact conical morphology with uniformly distributed particles and no apparent aggregation. AFM imaging revealed severe spherical aggregation in GCAPS-LS, whereas further analysis demonstrated that GCAPS-NS possessed significantly lower roughness values, indicative of smoother surfaces. However, the particle sizes of GCAPS-LS and GCAPS-NS determined by AFM and TEM were greater than those obtained through nanoparticle sizing. This discrepancy might be attributed to the misidentification of peptide aggregates as single particles in AFM and TEM imaging, which could lead to an overestimation of particle size due to the limited resolution of these techniques in distinguishing fine-scale aggregates.

FT-IR was further employed to investigate the interactions between GCAPS and the encapsulation system. As shown in Figure 2C, structural changes were observed after GCAPS encapsulation. In the GCAPS spectrum, the characteristic peaks at 3412 cm^−1^ and 2915.98 cm^−1^ corresponded to O-H stretching and symmetric -CH_2_ stretching vibrations, respectively. The blank liposomes and niosomes exhibited characteristic peaks at 2959.98 cm^−1^ and 2865.85 cm^−1^, which were attributed to β-sitosterol [1]. GCAPS displayed C=O stretching peaks at 1740 cm^−1^ and 1265 cm^−1^, with additional absorption bands at 1740 cm^−1^ and 1706 cm^−1^. For the liposomal formulations, the peak at 1740 cm^−1^ corresponded to ester C=O stretching in phospholipids, while the band at 1104 cm^−1^ was assigned to C-O stretching of Tween 40 [24]. The FT-IR spectra indicated slight structural alterations upon GCAPS encapsulation. In the GCAPS-loaded system, the peak intensity at 2865.85 cm^−1^ decreased, suggesting possible interactions between GCAPS and β-sitosterol that may facilitate encapsulation. Moreover, after GCAPS loading, minor shifts and intensity changes were observed in the amide I/II regions, indicating subtle alterations in the peptide microenvironment. The N-H stretching peak exhibited broadening and a slight red shift, suggesting hydrogen-bonding interactions between GCAPS and the lipid matrix, while minor changes in the C-H stretching region (~2850–2950 cm^−1^) indicated potential hydrophobic interactions. These interactions likely contributed to the successful encapsulation of GCAPS [14].

### 3.3. In Vitro Simulated Gastrointestinal Digestion Stability

**Figure 3:**
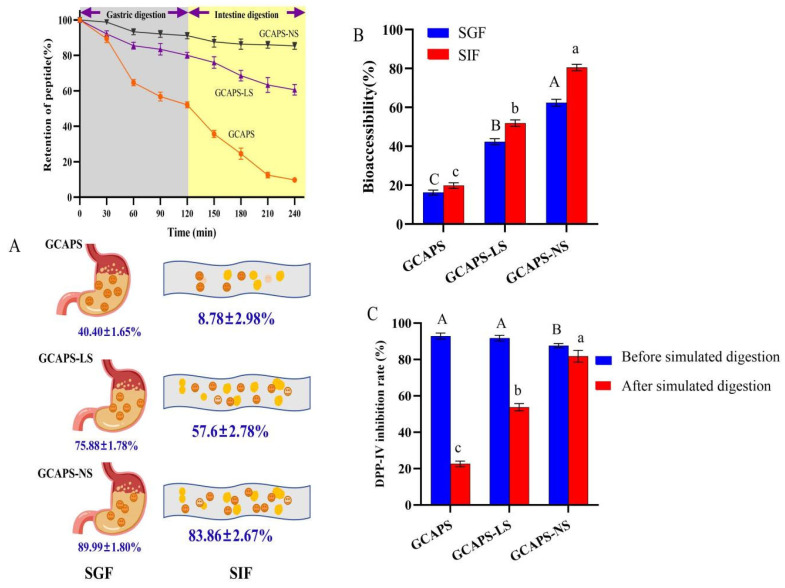
In vitro simulated release rate (**A**), bioaccessibility (**B**), and DPP-IV inhibition rates of GCAPS, GCAPS-LS, and GCAPS-NS before and after simulated gastric and intestinal digestion (**C**). (Different uppercase and lowercase letters indicate significant differences between groups. (*p* < 0.05). The data in the bar graphs are presented as mean ± standard deviation (SD).

Figure 3A showed that after 2 h of gastric digestion, the retention rates of free GCAPS, GCAPS-LS, and GCAPS-NS were 40.40 ± 1.65%, 75.88 ± 1.78%, and 83.86 ± 1.80%, respectively. These results suggested that both GCAPS-LS and GCAPS-NS effectively enhanced the gastric stability of GCAPS. Notably, the superior stability of GCAPS-NS might be attributed to the steric hindrance generated by the head groups of Tween-40. Following 2 h of simulated intestinal digestion, the retention rates of GCAPS, GCAPS-LS, and GCAPS-NS decreased to 8.78 ± 2.98%, 57.60 ± 2.78%, and 83.86 ± 2.67%, respectively. The bioaccessibility values were 19.8 ± 1.43% for GCAPS, 51.88 ± 1.67% for GCAPS-LS, and 80.44 ± 1.70% for GCAPS-NS (Figure 3B), indicating significantly enhanced bioaccessibility of GCAPS when encapsulated in a nanoparticle system. As shown in Figure 3C, the DPP-IV inhibition rates of GCAPS, GCAPS-LS, and GCAPS-NS before digestion were 92.87 ± 1.67%, 91.74 ± 1.56%, and 87.65 ± 1.11%, respectively. After gastrointestinal digestion, the DPP-IV inhibitory activities of GCAPS, GCAPS-LS, and GCAPS-NS were 22.60 ± 1.54%, 53.76 ± 2.01%, and 81.79 ± 3.22%, respectively, indicating that GCAPS-NS retained significantly higher DPP-IV inhibitory activity under digestive conditions (Figure 3C). These findings confirmed that GCAPS-NS significantly enhances the digestive stability and hypoglycemic bioactivity of GCAPS. However, as in vitro digestion models might not fully replicate in vivo conditions, further animal experiments were conducted to verify the actual bioavailability of GCAPS-NS.

### 3.4. Effects of GCAPS and GCAPS-NS Intervention on Fat Accumulation and Body Weight Gain in Mice

To further evaluate the hypoglycemic effects of GCAPS and GCAPS-NS in vivo, mice were randomly assigned to nine groups, with eight animals per group. The experimental groups were as follows: NCD was the normal control diet group; HFD was the high-fat diet group; GCAPS was the high-fat diet group with GCAPS intervention; HFD+NSL was the high-fat diet group with a low peptide dose; HFD+NSM was the high-fat diet group with a medium peptide dose; and HFD+NSH was the high-fat diet group with a high peptide dose.

**Figure 4:**
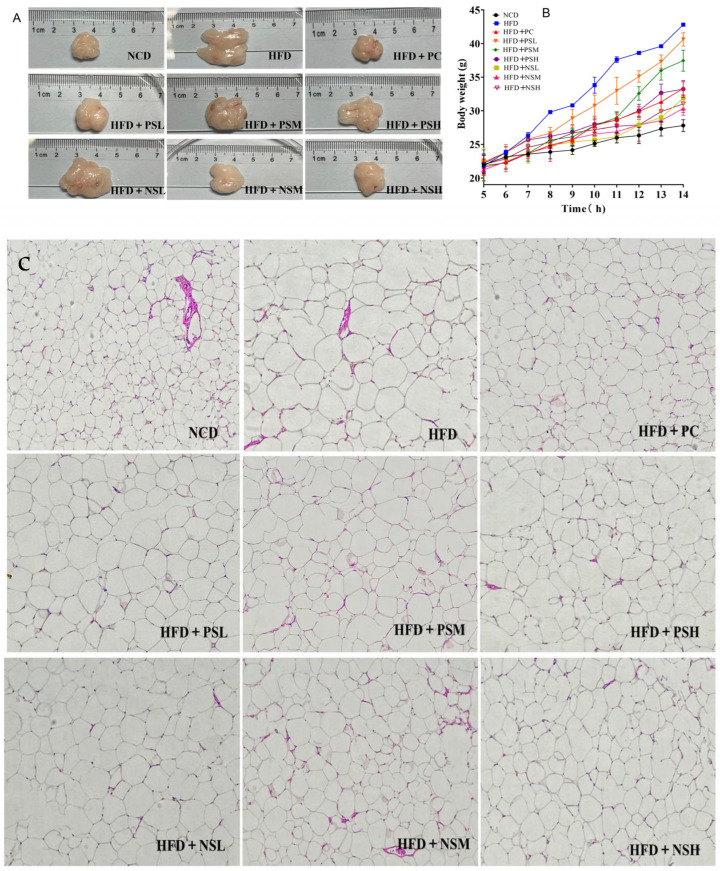
Effects of GCAPS and different doses of GCAPS-NS on fat accumulation and body weight gain in mice (**A**,**B**); HE staining of epididymal adipose tissue in mice from the GCAPS and different-dose GCAPS-NS groups (**C**).

As shown in Figure 4A, epididymal adipose tissue from the HFD group exhibited significantly greater fat deposition compared to other groups. In addition, fat deposition in the GCAPS group was higher than that in the GCAPS-NS group, which might suggest that GCAPS-NS was more effective in reducing adiposity. The body weight gain curve further revealed a progressively widening gap between the HFD and the NCD group over time. Figure 4C showed that the HFD group exhibited substantial epididymal fat accumulation accompanied by enlarged adipocyte size. Compared with the HFD group, the GCAPS intervention group showed a modest reduction in adiposity. Specifically, mice in the HFD+NSM and HFD+NSH groups exhibited a marked decrease in adipocyte vacuolization.

### 3.5. Effects of GCAPS and GCAPS-NS Intervention on White and Brown Adipose Tissue Indices in Mice

Table 1 showed that the epididymal and perirenal fat indices were significantly increased in the HFD group compared to the NCD group (*p* < 0.05), indicating excessive fat accumulation. The epididymal fat index was reduced by 32.29%, 23.96%, 26.00%, 28.25%, 32.18%, 33.96%, and 32.51% in the HFD+PC, HFD+PSL, HFD+PSM, HFD+PSH, HFD+NSL, HFD+NSM, and HFD+NSH groups, respectively. Similarly, the perirenal fat index decreased by 25.30%, 15.56%, 17.97%, 21.72%, 34.62%, 48.26%, and 51.95% in the corresponding groups. Brown adipose tissue (BAT) index is rich in capillaries and mitochondria, and plays a crucial role in accelerating metabolism. It promotes the breakdown of intracellular fatty acids through uncoupled oxidative phosphorylation, thereby generating heat and stimulating the consumption of white adipose tissue. The BAT index was significantly lower in the HFD group compared to the NCD group, indicating that HFD impairs the normal development and growth of BAT in mice. After intervention with GCAPS or GCAPS-NS, the BAT index showed a significant increase, with values in the GCAPS-NS group nearly restored to those of the NCD group. These results suggested that GCAPS-NS effectively mitigates HFD-induced BAT underdevelopment, thereby contributing to obesity prevention and the maintenance of glucose homeostasis.

### 3.6. Effects of GCAPS and GCAPS-NS Intervention on Serum Lipid and Hepatic Lipid Levels in Mice

**Figure 5:**
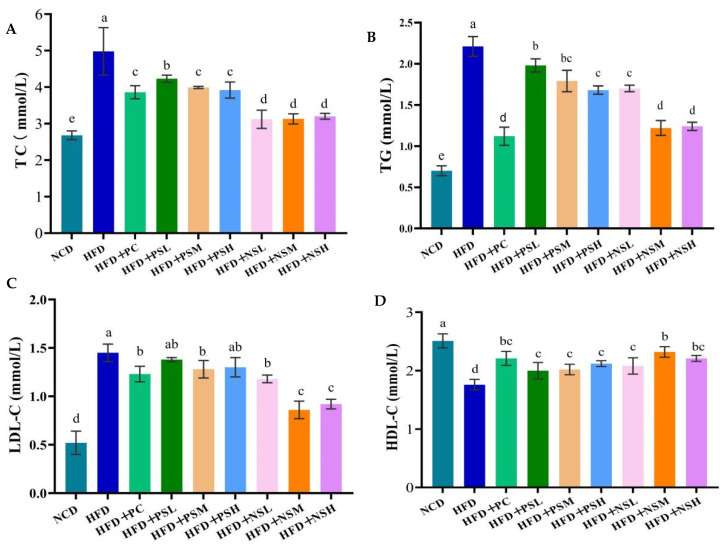
Effects of GCAPS and different doses of GCAPS-NS on blood lipid (**A**,**B**) and hepatic lipid (**C**,**D**) levels in mice. Different lowercase letters indicate significant differences between groups (*p* < 0.05). The data in the bar graphs are presented as mean ± standard deviation (SD).

As shown in Figure 5A,B, HFD group significantly elevated serum TC and TG levels (*p* < 0.05). GCAPS and GCAPS-NS interventions significantly decreased TC levels (*p* < 0.05), with the greatest reduction observed in the HFD+NSM group. Compared with the HFD group, serum TC levels in the HFD+PC, HFD+PSL, HFD+PSM, HFD+PSH, HFD+NSL, HFD+NSM, and HFD+NSH groups were reduced by 22.48%, 15.06%, 19.88%, 21.28%, 38.15%, 39.36%, and 38.75%, respectively. Moreover, serum TG levels in these groups decreased by 49.32%, 10.41%, 19.00%, 23.98%, 23.08%, 44.79%, and 44.34%, respectively. The HFD group exhibited significantly elevated LDL-C levels compared to the NCD group (*p* < 0.05). However, intervention with PC, GCAPS, or GCAPS-NS significantly reduced LDL-C levels (*p* < 0.05), with the largest decrease observed in the HFD+NSM group. Moreover, HFD feeding significantly decreased HDL-C levels, whereas interventions with PC, GCAPS, or GCAPS-NS significantly increased HDL-C levels (*p* < 0.05) (Figure 5C,D). These results suggested that GCAPS-NS effectively improves lipid metabolism and alleviates hepatic damage induced by long-term high-fat diet consumption.

### 3.7. Effects of GCAPS and GCAPS-NS Intervention on Liver Tissue in Mice

**Figure 6:**
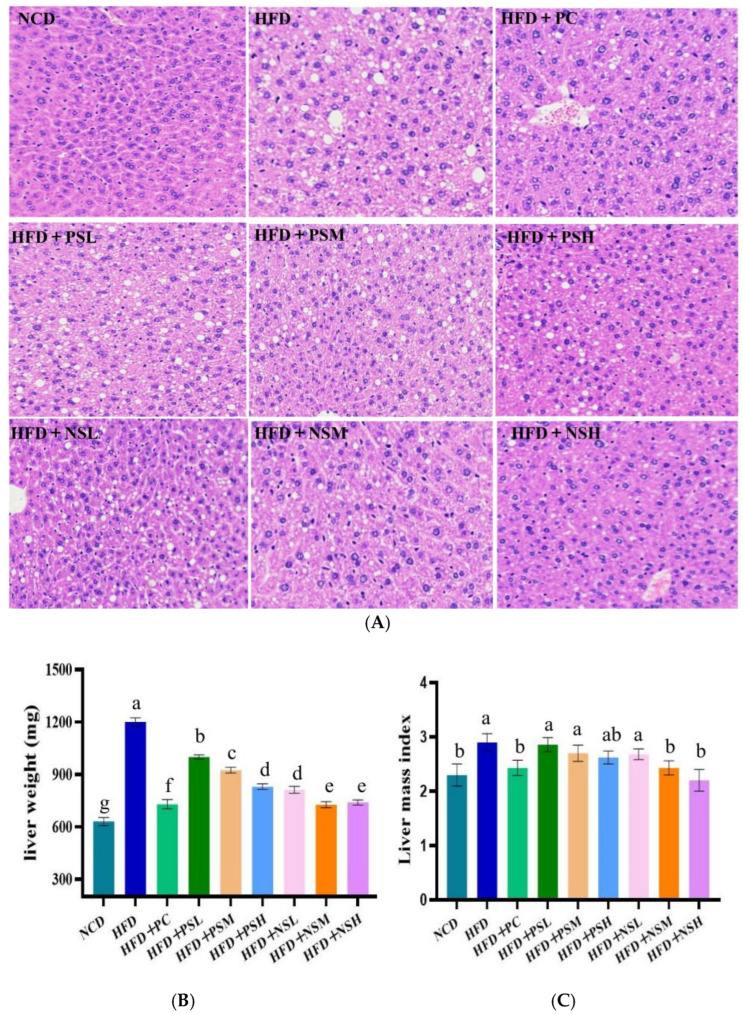
Effects of GCAPS and different doses of GCAPS-NS on the pathological morphology (**A**) and liver index (**B**) of mouse liver tissue (**C**). Different lowercase letters indicate significant differences between groups (*p* < 0.05). The data in the bar graphs are presented as mean ± standard deviation (SD).

As shown in Figure 6A, histological analysis of liver tissue revealed severe hepatic steatosis in the HFD group, characterized by hepatocellular enlargement, disorganized cellular architecture, extensive intracellular lipid vacuolization, and partial nuclear pyknosis, indicating that HFD induced marked lipid degeneration in the liver. Prominent lipid vacuolization remained evident in hepatocytes of the HFD+PSL and HFD+PSM groups, suggesting that the reduced efficacy of GCAPS might be associated with its instability and degradation under physiological conditions. GCAPS-NS intervention alleviated hepatocellular vacuolization, with particularly pronounced improvements observed in the HFD+NSM and HFD+NSH groups. These results suggested that GCAPS-NS effectively protected GCAPS from degradation and enhanced its biological efficacy in vivo.

Liver weight and index were lower in the NCD group than in the HFD group, suggesting that the latter experienced more severe hepatic lipid accumulation and injury. These findings suggested that GCAPS-NS effectively protected GCAPS from degradation and enhanced its biological efficacy in vivo. In addition, liver weight and index were significantly reduced in the NCD group relative to the HFD group, indicating that HFD consumption induced hepatic lipid accumulation and injury. After interventions with PC, GCAPS, and GCAPS-NS, hepatic fat accumulation was significantly reduced, especially in the PC and GCAPS-NS groups (Figure 6B). Compared to the HFD group, GCAPS treatment moderately reduced the liver index, whereas all GCAPS-NS intervention groups attenuated hepatic injury, with no significant differences observed between the HFD+NSM, HFD+NSH, and PC groups (Figure 6C).

### 3.8. Effects of GCAPS and GCAPS-NS Intervention on GTT and ITT

**Figure 7:**
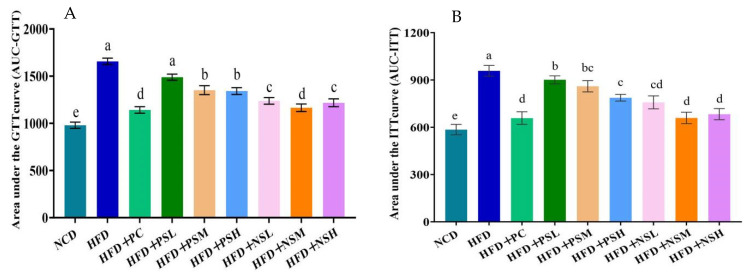
Effects of GCAPS and GCAPS-NS on glucose tolerance (**A**) and insulin tolerance (**B**) in mice. Different lowercase letters indicate significant differences between groups (*p* < 0.05). The data in the bar graphs are presented as mean ± standard deviation (SD).

As shown in Table 2 and Table 3 and Figure 7A,B, HFD-fed mice displayed impaired glucose tolerance, evidenced by delayed blood glucose clearance and a significantly larger area under the GTT curve (*p* < 0.05). Intervention with PC and GCAPS-NS significantly improved glucose tolerance in HFD-fed mice. As shown by insulin tolerance testing, the HFD group exhibited impaired glucose clearance, with minimal reduction in blood glucose levels within 60 min post-injection and a rapid rebound thereafter, indicative of insulin resistance. The HFD+NSM group exhibited greater improvement in insulin sensitivity compared to the HFD+NSH group. To investigate this phenomenon, the intestinal absorption and permeability of niosome formulations at different doses were evaluated using an Ussing chamber. As shown in Figure S4, the apparent Papp, Er, and particle size of HFD+NSL, HFD+NSM, and HFD+NSH in rat colonic mucosa were compared. Significant differences in Papp were observed among the three groups in both mucosal-to-serosal (M-S) and serosal-to-mucosal (S-M) directions (*p* < 0.05). HFD+NSM exhibited a markedly lower Er compared with HFD+NSL and HFD+NSH. Notably, HFD+NSH showed an increased particle size relative to HFD+NSM, likely due to higher peptide loading, which promotes a more densely packed internal structure and particle aggregation. The enlarged particle size of HFD+NSH reduced fusion efficiency with the colonic mucosa, resulting in a higher Er and lower absorption efficiency. Moreover, elevated peptide content may alter niosome membrane fluidity or surface properties, further affecting mucosal retention and transport.

### 3.9. Effects of GCAPS and GCAPS-NS Intervention on Serum Glucose, hsCRP, and GLP-1 Levels in Mice

**Figure 8:**
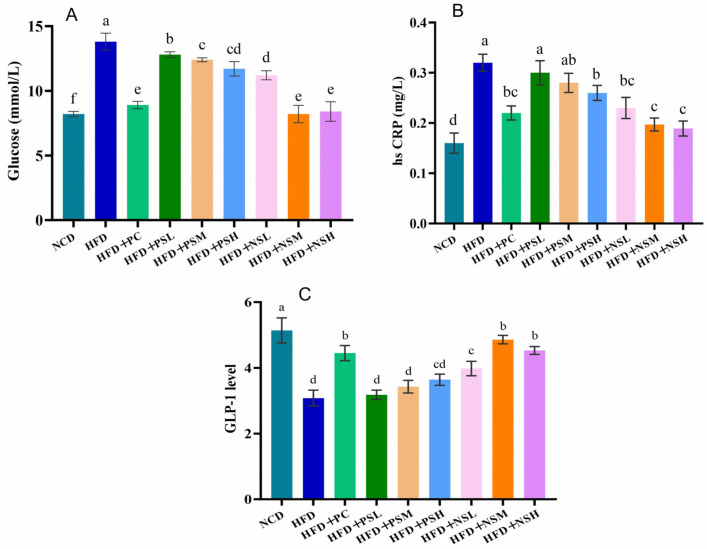
Effects of GCAPS and GCAPS-NS on mouse serum glucose, hsCRP and GLP-1 level (**A**–**C**). Different lowercase letters indicate significant differences between groups (*p* < 0.05). The data in the bar graphs are presented as mean ± standard deviation (SD).

Figure 8A–C demonstrated that HFD induced a significant rise in serum glucose and hsCRP levels relative to the NCD group (*p* < 0.05). Both serum glucose and hsCRP levels showed a dose-dependent decline in the HFD+NSM and HFD+NSH groups, indicating that GCAPS-NS effectively preserved peptide integrity and improved glycemic control. GLP-1 analysis indicated a significant decrease in serum GLP-1 concentrations in HFD-fed mice relative to the NCD group (*p* < 0.05). HFD+NSM intervention significantly increased GLP-1 levels compared to the HFD group, particularly in the HFD+NSM group. This might be attributed to the ability of HFD+NSM to protect GCAPS from degradation and promote GLP-1 secretion. Considering that DPP-IV catalyzes the rapid inactivation of circulating GLP-1, the elevated GLP-1 concentrations observed in GCAPS-NS–treated mice may reflect attenuation of DPP-IV–mediated degradation or enhanced peptide stability. This interpretation provides mechanistic insight into how GCAPS-NS could sustain GLP-1 bioactivity and improve glucose regulation, although direct measurement of DPP-IV activity is required for confirmation.

Based on the above results, the effects of high-dose HFD+PSH and HFD+NSM on gut microbiota composition were further evaluated.

### 3.10. Effects of GCAPS and GCAPS-NS Intervention on Gut Microbiota Composition in Mice

**Figure 9:**
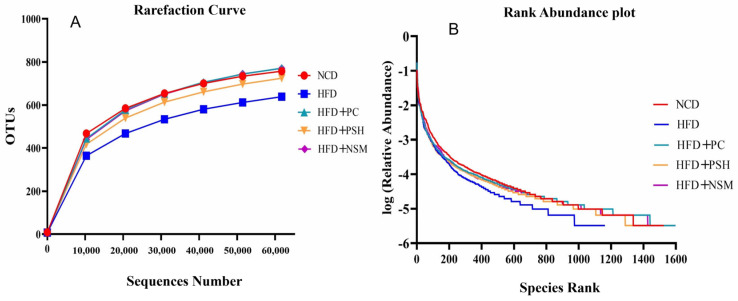
Dilution curves and Rank Abundance curves of different sample treatment groups (**A**,**B**).

**Figure 10:**
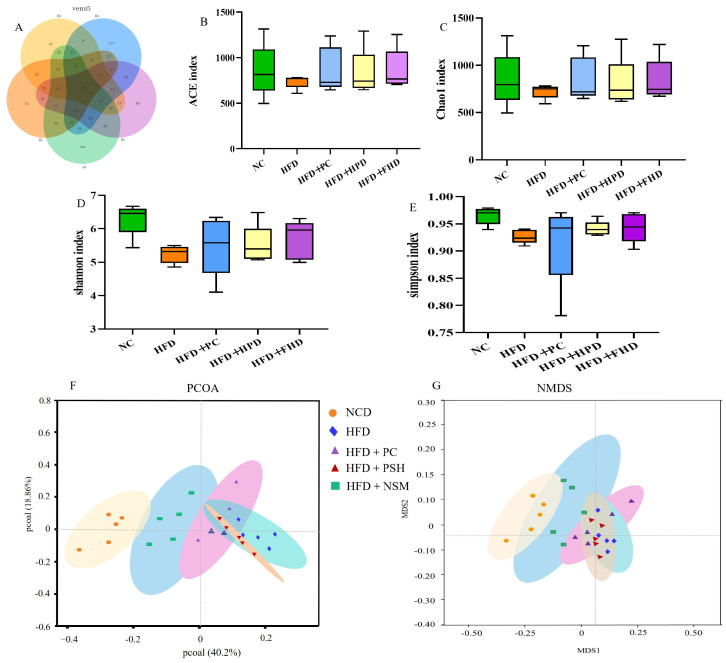
The intestinal microbes of mice in each group. Venn diagram of gut microbiota OTUs in the blank control (NCD), high-fat diet (HFD), positive control (HFD+PC), high-dose peptide group (HFD+PSH), and medium-dose peptide liposome group (HFD+NSM) (**A**); ACE index (**B**); Chao index (**C**); Shannon index (**D**); Simpson index (**E**); PCOA scatter diagram (**F**); NMDS scatter diagram (**G**). For panels (**B**–**G**), *n* = 5 mice per group. Statistical analysis was performed using one-way ANOVA followed by Tukey’s post hoc test.

As shown in Figure 9A, the rarefaction curves of all five groups approached a plateau with increasing sequencing depth, suggesting sufficient coverage for robust microbial diversity assessment. The rank abundance curves further demonstrated broadly and evenly distributed microbial communities across all groups, except for the HFD group, in which a steep decline in the curve indicated a marked reduction in species richness and evenness (Figure 9B). These results indicated that HFD significantly impaired gut microbial diversity and structure. Venn diagram analysis revealed 802 shared OTUs across the five groups, with the HFD+PC (177) and HFD+NSM (162) groups showing the greatest uniqueness, suggesting improved microbial diversity following intervention (Figure 10A). Compared to the NCD group, the HFD group exhibited significantly reduced ACE, Chao1, Simpson, and Shannon indices (*p* < 0.05), suggesting that HFD impaired microbial richness and diversity [24]. However, the Shannon index was significantly increased in the HFD+PC and HFD+NSM groups, approaching the levels observed in the NCD group (Figure 10B–E. PCoA and NMDS analyses illustrated significant divergence in microbial profiles between HFD-fed mice and those receiving dietary interventions [25]. Among them, the HFD+NSM group exhibited a distinct shift away from the HFD group along both the PCoA (PC1 and PC2) and NMDS (MDS1 and MDS2) dimensions, with its microbial profile clustering more closely with that of the NCD group (Figure 10F,G. These findings suggested that PSH and NSM interventions modulated the gut microbiota composition in the context of HFD-induced dysbiosis, with HFD+NSM exhibiting a more pronounced regulatory effect.

### 3.11. Effects of Different Intervention Groups on Gut Microbial Communities in Mice

**Figure 11:**
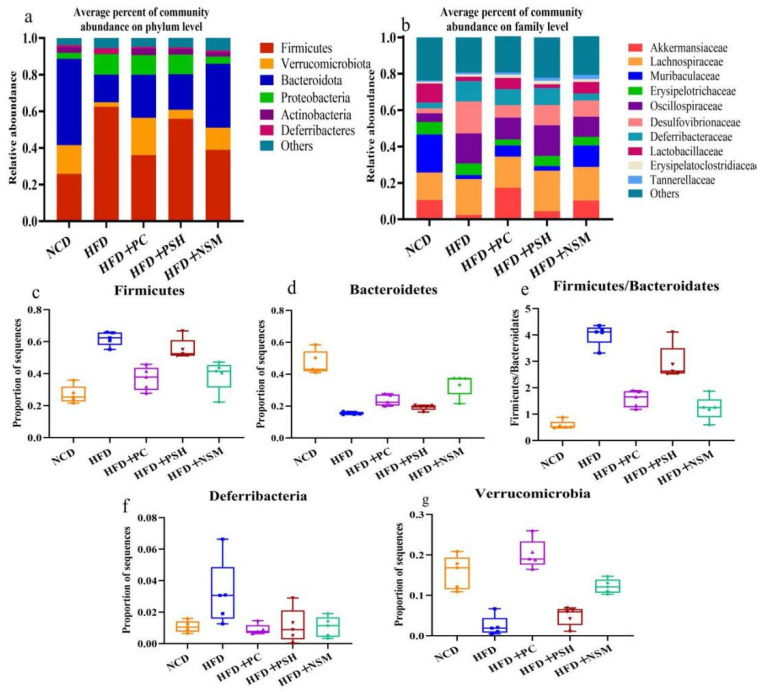
Effect of the level of intestinal flora in each group of mice (**a**). Effects of different sample interventions on the level of intestinal flora in HFD mice (**b**). Relative abundance of *Firmicutes* (**c**), Bacteroidetes (**d**), *Firmicutes*/*Bacteroidetes* abundance ratio (**e**), *Verrucomicrobia* (**f**), and Deferribacteria (**g**) in fecal microflora of different sample intervention groups. For panels (**c**–**g**), *n* = 5 mice per group. Statistical analysis was performed using one-way ANOVA followed by Tukey’s post hoc test.

Microbial imbalance in the gut has been closely related to the development of metabolic diseases, including diabetes [26]. As shown in Figure 11a, HFD led to reduced abundance of *Bacteroidetes* and *Verrucomicrobia*, and elevated levels of *Firmicutes* and *Desulfovibrionaceae*, compared to the NCD group. At the phylum level, *Firmicutes* and *Bacteroidetes* comprised the principal bacterial phyla observed across all groups (Figure 11b). In the NCD group, *Bacteroidetes* was predominant, whereas *Firmicutes* became the dominant phylum in the HFD group. It was observed that HFD feeding led to an enrichment of *Firmicutes* and *Proteobacteria*, while reducing that of *Bacteroidetes* and *Verrucomicrobia*. Compared with the HFD group, the HFD+PC, HFD+PSH, and HFD+NSM intervention groups exhibited a reduced *Firmicutes* dominance and a higher proportion of *Bacteroidetes*. At the family level, the NCD group was dominated by *Akkermansiaceae*, *Muribaculaceae*, *Lachnospiraceae*, and *Lactobacillaceae*. *Akkermansiaceae* abundance was significantly elevated in both the HFD+PC and HFD+NSM groups. Moreover, all three intervention groups alleviated gut microbial dysbiosis associated with insulin resistance by reducing the *Firmicutes*/*Bacteroidetes* ratio (Figure 11c–g). Among them, the HFD+NSM group exerted the most pronounced modulatory effect [27].

### 3.12. Effects of Different Intervention Groups on the Gut Microbiota Structure in Mice

**Figure 12:**
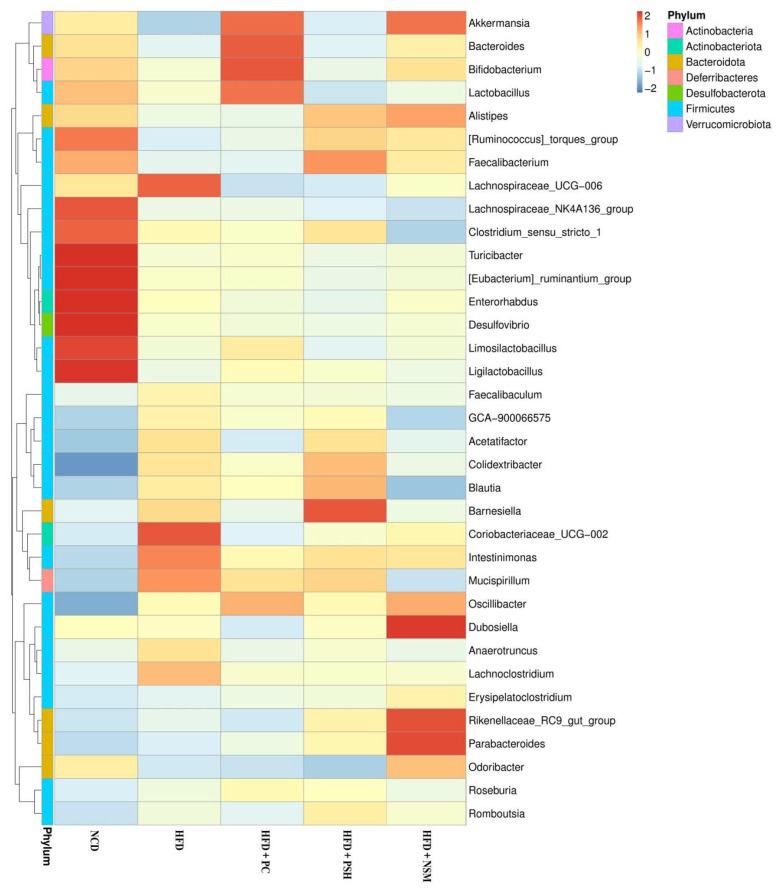
Heat map of the composition of mouse gut microbiota at the genus level.

Figure 12 showed that the NCD group exhibited increased levels of functionally beneficial genera, including *Lachnospiraceae_NK4A136_group*, *Limosilactobacillus*, and *Turicibacter*, which contribute to metabolic regulation, immune enhancement, and fermentation [28,29,30]. In the HFD group, the abundances of *Lachnoclostridium*, *Mucispirillum*, and *Intestinimonas* were increased, suggesting that the high-fat diet induced gut microbiota dysbiosis. Following intervention, the abundances of *Akkermansia*, *Bacteroides*, and *Bifidobacterium* were increased in the HFD+PC and HFD+NSM groups. Among these, *Akkermansia* was known to improve insulin resistance and modulate immune responses [31]. The HFD+NSM group was also enriched in beneficial genera such as *Parabacteroides* and *Dubosiella*, among which *Parabacteroides* has been negatively associated with metabolic disorders [32]. In addition, the HFD+PSH group exhibited elevated levels of SCFA-producing microbes, including *Barnesiella* and *Faecalibacterium*. The HFD+PC, HFD+PSH, and HFD+NSM interventions all alleviated HFD-induced gut microbiota dysbiosis, with the HFD+NSM group exhibiting the most pronounced regulatory effect, potentially contributing to the maintenance of glucose homeostasis.

**Figure 13:**
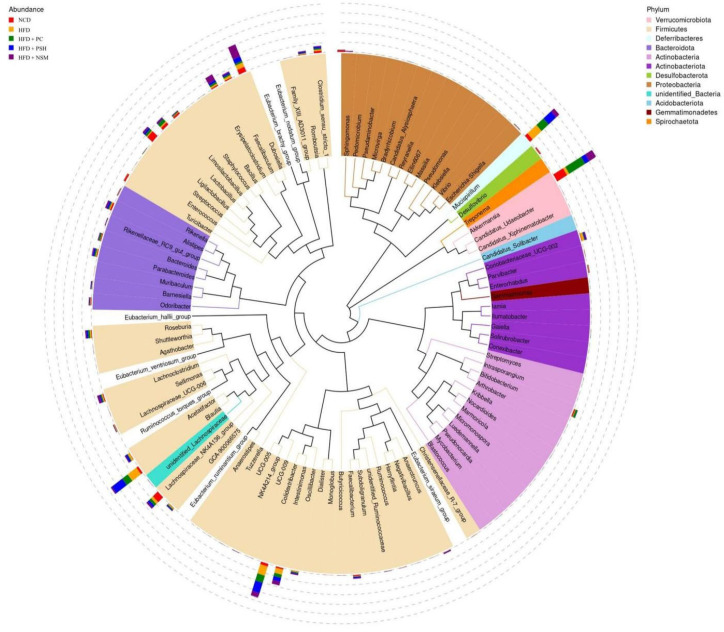
Phylogenetic relationships of gut microbiota at the genus level in mice from different sample intervention groups. Different colors represent different phylum-level classifications.

As shown in the genus-level phylogenetic tree in Figure 13, microbial diversity was reduced in the HFD group, accompanied by decreased abundances of *Akkermansia* and *Lactobacillus*, and increased abundances of *Blautia* and *Colidextribacter*. Additionally, *Akkermansia* has been shown to enhance mucosal integrity and reduce inflammation, and might have contributed to the management of type 2 diabetes [33]. *Lactobacillus* was considered a probiotic that protected the intestinal barrier and improved gut permeability, which might be associated with enhanced glucose tolerance, glucose-stimulated insulin secretion, and reduced inflammation in the host [34]. Compared with the HFD group, the HFD+NSM group showed increased abundances of *Akkermansia*, *Lactobacillus*, and *Dubosiella*, suggesting that GCAPS-NS intervention might have exerted a positive effect on the improvement of type 2 diabetes.

## 4. Conclusions

In this study, GCAPS-loaded nanoparticle delivery systems (GCAPS-LS and GCAPS-NS) were successfully developed. The successful construction of the nanoparticles was confirmed by morphological characterization and FT-IR spectroscopy. In vitro simulated gastrointestinal digestion experiments demonstrated that GCAPS-NS exhibited favorable digestive stability and potent hypoglycemic activity. Furthermore, an HFD-induced insulin resistance mouse model was employed to systematically evaluate the in vivo hypoglycemic effects of GCAPS and GCAPS-NS, as well as their regulatory impact on gut microbiota. Compared to GCAPS, GCAPS-NS demonstrated superior efficacy in alleviating hepatic steatosis and lowering serum levels of TC, TG, and LDL-C. GCAPS-NS significantly improved glucose metabolism by enhancing glucose tolerance and insulin sensitivity, preserving pancreatic islet integrity, and reducing circulating hsCRP levels. In addition, GCAPS-NS promoted the secretion of endogenous GLP-1, thereby contributing to the maintenance of glucose homeostasis, particularly in the HFD+NSM group. Moreover, gut microbiota analysis revealed that the HFD+NSM intervention effectively alleviated high-fat diet-induced dysbiosis, as evidenced by a reduced *Firmicutes*/*Bacteroidetes* ratio and an increased abundance of *Akkermansia*. These findings suggested that GCAPS-NS significantly improves in vivo stability and holds promise as a therapeutic strategy for managing insulin resistance and type 2 diabetes via metabolic regulation and modulation of gut microbiota.

## Figures and Tables

**Table 1:** Effects of white fat index and brown fat index on mice in each group.

Samples	Epididymal Fat Index	Perirenal Fat Index	Brown Adipose Tissue Index	Inguinal Fat Index
NCD	14.65 ± 1.65 ^d^	4.69 ± 0.03 ^e^	5.55 ± 0.33 ^a^	6.07 ± 0.02 ^f^
HFD	53.88 ± 1.89 ^a^	18.69 ± 0.98 ^a^	2.63 ± 0.58 ^h^	28.31 ± 0.03 ^a^
HFD+PC	36.48 ± 2.02 ^bc^	13.96 ± 0.76 ^c^	3.94 ± 0.05 ^d^	13.77 ± 0.05 ^d^
HFD+PSL	40.97 ± 1.33 ^b^	15.78 ± 1.01 ^b^	3.78 ± 0.01 ^e^	19.59 ± 0.04 ^b^
HFD+PSM	39.87 ± 1.95 ^bc^	15.33 ± 1.34 ^bc^	3.32 ± 0.03 ^g^	18.77 ± 1.01 ^b^
HFD+PSH	38.66 ± 2.01 ^bc^	14.63 ± 0.54 ^bc^	3.42 ± 0.02 ^f^	17.45 ± 1.21 ^bc^
HFD+NSL	36.54 ± 1.62 ^bc^	12.22 ± 1.38 ^cd^	4.02 ± 0.05 ^d^	15.43 ± 1.34 ^c^
HFD+NSM	35.58 ± 1.15 ^c^	9.67 ± 2.45 ^d^	4.54 ± 0.01 ^b^	13.23 ± 0.21 ^e^
HFD+NSH	36.36 ± 1.01 ^c^	8.98 ± 1.05 ^d^	4.31 ± 0.05 ^c^	13.54 ± 0.51 ^d^

^a,b,c,d,e,f,g,h^ Different lowercase letters indicate significant differences between groups (*p* < 0.05).

**Table 2:** Effects of GCAPS and GCAPS-NS on glucose tolerance in mice.

Samples	0 min	30 min	60 min	90 min	120 min
NCD	5.8 ± 0.02 ^eF^	10.8 ± 0.07 ^aH^	10.0 ± 0.13 ^bH^	8.2 ± 0.21 ^cI^	6.4 ± 0.04 ^dH^
HFD	8.6 ± 0.23 ^eA^	17.8 ± 0.12 ^aA^	16.7 ± 0.08 ^bA^	15.8 ± 0.01 ^cA^	13.3 ± 0.23 ^dA^
HFD+PC	7.9 ± 0.02 ^eC^	16.4 ± 0.14 ^aB^	15.2 ± 0.08 ^bB^	14.0 ± 0.12 ^cB^	10.8 ± 0.20 ^dC^
HFD+PSL	8.2 ± 0.23 ^eAB^	15.6 ± 0.14 ^aC^	13.6 ± 0.08 ^bC^	11.8 ± 0.04 ^cE^	10.9 ± 0.12 ^dC^
HFD+PSM	8.3 ± 0.16 ^eAB^	14.8 ± 0.10 ^aD^	13.4 ± 0.07 ^bD^	12.2 ± 0.06 ^cD^	10.2 ± 0.02 ^dD^
HFD+PSH	7.9 ± 0.12 ^eB^	13.7 ± 0.05 ^aE^	13.0 ± 0.03 ^bF^	12.6 ± 0.12 ^cC^	12.0 ± 0.16 ^dB^
HFD+NSL	7.4 ± 0.13 ^eE^	12.2 ± 0.08 ^bF^	13.3 ± 0.17 ^aD^	10.6 ± 0.11 ^cF^	9.8 ± 0.08 ^dE^
HFD+NSM	7.8 ± 0.03 ^dD^	10.6 ± 0.02 ^bI^	12.4 ± 0.14 ^aG^	9.8 ± 0.06 ^cG^	7.8 ± 0.14 ^dG^
HFD+NSH	8.1 ± 0.08 ^eBC^	11.6 ± 0.12 ^bG^	13.2 ± 0.10 ^aE^	9.4 ± 0.05 ^cH^	8.3 ± 0.02 ^dF^

^a,b,c,d,e^ Different lowercase letters indicate significant differences within the same row (*p* < 0.05). ^A,B,C,D,E,F,G,H,I^ Different uppercase letters indicate significant differences within the same column (*p* < 0.05).

**Table 3:** Effects of GCAPS and GCAPS-NS on insulin tolerance in mice.

Samples	0 min	30 min	60 min	90 min	120 min
NCD	8.00 ± 0.01 ^aC^	5.80 ± 0.02 ^bG^	4.30 ± 0.03 ^eH^	4.86 ± 0.02 ^dI^	5.00 ± 0.01 ^cG^
HFD	9.90 ± 0.02 ^aA^	8.80 ± 0.12 ^dA^	8.50 ± 0.13 ^eA^	9.37 ± 0.07 ^bA^	8.96 ± 0.02 ^cA^
HFD+PC	8.23 ± 0.04 ^aB^	6.89 ± 0.04 ^bF^	6.22 ± 0.12 ^dF^	5.83 ± 0.04 ^eB^	6.65 ± 0.14 ^cB^
HFD+PSL	8.34 ± 0.23 ^aB^	7.88 ± 0.02 ^bB^	6.90 ± 0.08 ^cB^	6.65 ± 0.04 ^dE^	6.58 ± 0.12 ^dB^
HFD+PSM	8.00 ± 0.03 ^aC^	7.67 ± 0.12 ^bCD^	6.75 ± 0.04 ^cC^	6.28 ± 0.10 ^dD^	6.10 ± 0.06 ^eC^
HFD+PSH	7.89 ± 0.02 ^aD^	7.53 ± 0.11 ^bCD^	6.56 ± 0.03 ^cD^	5.84 ± 0.12 ^dC^	5.43 ± 0.06 ^eD^
HFD+NSL	7.80 ± 0.04 ^aC^	7.65 ± 0.02 ^bC^	6.44 ± 0.05 ^cE^	5.43 ± 0.10 ^dF^	5.38 ± 0.14 ^dD^
HFD+NSM	8.01 ± 0.13 ^aC^	7.12 ± 0.07 ^bE^	5.68 ± 0.12 ^cG^	5.11 ± 0.03 ^eG^	5.21 ± 0.02 ^dF^
HFD+NSH	8.10 ± 0.09 ^aBC^	7.44 ± 0.12 ^bD^	6.23 ± 0.11 ^cF^	5.34 ± 0.06 ^cH^	5.28 ± 0.02 ^dE^

^a,b,c,d,e^ Different lowercase letters indicate significant differences within the same row (*p* < 0.05). ^A,B,C,D,E,F,G,H,I^ Different uppercase letters indicate significant differences within the same column (*p* < 0.05).

## Data Availability

The original contributions presented in this study are included in the article/Supplementary Materials. Further inquiries can be directed to the corresponding authors.

## Supplementary Materials

The following supporting information can be downloaded at: https://www.mdpi.com/article/10.3390/foods14213795/s1, Figure S1: Visualization (a and b), encapsulation efficiency, particle size (c and f), ζ-potential, PDI (d and g) and particle size distribution (e and h) of GCAPS liposomes and niosomes; Figure S2: Single factor experiments for the preparation of liposomes and niosomes loaded with GCAPS (A–E); Figure S3: Visualization of GCAPS encapsulation systems stored at different temperature (4 °C, 25 °C, and 37 °C), pH (2, 4, 6, 8) and NaCl concentration (30, 90, 150, 250 mM); Figure S4: Total ion chromatograms of GCAP molecular weight <3 kDa ultrafiltration fractions; Figure S5: Sephadex G-15 gel chromatogram of GCAP and GWPP (A and E). IC50 values for DPP-IV, α-amylase and α-glucosidase in fractions GCAP-(S1–S4), RP-HPLC profile of partial GCAP-P1 (C and G). IC50 values for DPP-IV, α-amylase and α-glucosidase in fractions GCAP-S3-P1 (D1–D3 and H1–H3); Bars with different letters are significantly different at *p* < 0.05. Figure S6. Particle size, apparent permeability parameters, and efflux transport rates of GCAPS-NS at equivalent peptide doses (HFD+NSL, HFD+NSM, and HFD+NSH); Table S1. Sequence identification, docking energies, and DPP-IV-inhibitory IC50 values of DPP-IV-inhibitory peptides in GCAPS and GWPPG fractions.
