# Supplementary material for: Preparation, Characterization, and Mechanism of Hypoglycemic Action of a Goat Casein Peptide Delivery System Involving DPP-IV Inhibition and GLP-1 Release
Source: Foods. 2025 Nov 5;14(21):3795. doi: 10.3390/foods14213795 (PMC12610973; doi:10.3390/foods14213795)
Supplement: Supplementary file 1 [file foods-14-03795-s001.zip › foods-3898316-supplementary.pdf]

Supplementary Material

1.Materials and methods

1.1 Gel filtration chromatography of goat casein hydrolysate（GCA）

Separation was performed using a Sephadex G-15 gel filtration column (1.5 × 50 cm, 35 cm bed height) pre-equilibrated with Tris-acetate buffer (pH 7.0). The sample (20 mg/mL, 4 mL) was loaded and eluted with ultrapure water at 0.5 mL/min. Fractions were collected based on UV absorbance at 280 nm, and their DPP-IV inhibitory activities were measured. The most active fraction was further purified by reverse-phase high-performance liquid chromatography (RP-HPLC).

1.2. Purification of GCA Hydrolysate by Reverse-Phase High-Performance Liquid Chromatography (RP-HPLC)

The collected fractions were lyophilized and reconstituted to a concentration of 20 mg/mL, followed by filtration through a 0.45 μm membrane. The resulting solution was subjected to a second round of purification using preparative reverse-phase high-performance liquid chromatography (RP-HPLC). Chromatographic separation was performed on a CST C1 column (8–10 μm, 120 Å, 21.2 mm i.d. × 250 mm) with a sample injection volume of 500 μL and a flow rate of 10 mL/min. The mobile phase consisted of solvent A (ultrapure water containing 0.1% trifluoroacetic acid) and solvent B (acetonitrile containing 0.1% trifluoroacetic acid). The elution program was as follows: 0% B (0–5 min), 0–60% B (5–25 min), and 60–0% B (25–30 min). Eluted fractions corresponding to distinct UV peaks were collected, concentrated using rotary evaporation at 45 °C, freeze-dried, and stored at 4 °C for further analysis. The purified peptides were designated as GCAPS.

1.3. Analysis of gut microbiota in mice

The entire intestinal tract of mice was excised, and the cecum was aseptically isolated. Cecal contents were collected under sterile conditions and transferred into sterile centrifuge tubes. Bacterial genomic DNA was promptly extracted using a commercial kit. The V3-V4 region of the

16S rRNA gene was amplified from the bacterial genome to construct a short-read sequencing library. Paired-end sequencing was performed on the Illumina NovaSeq platform. Raw reads were merged, quality-filtered, and denoised to obtain high-quality sequences. Taxonomic annotation and abundance analysis were then conducted based on the resulting effective sequences to characterize the microbial composition of each sample. To assess variation in microbial composition among samples, α- and β-diversity analyses were further performed.

2. Results and discussion

2.1 Preparation of GCAPS-Loaded nanoparticle systems (GCAPS-LS and GCAPS-NS)

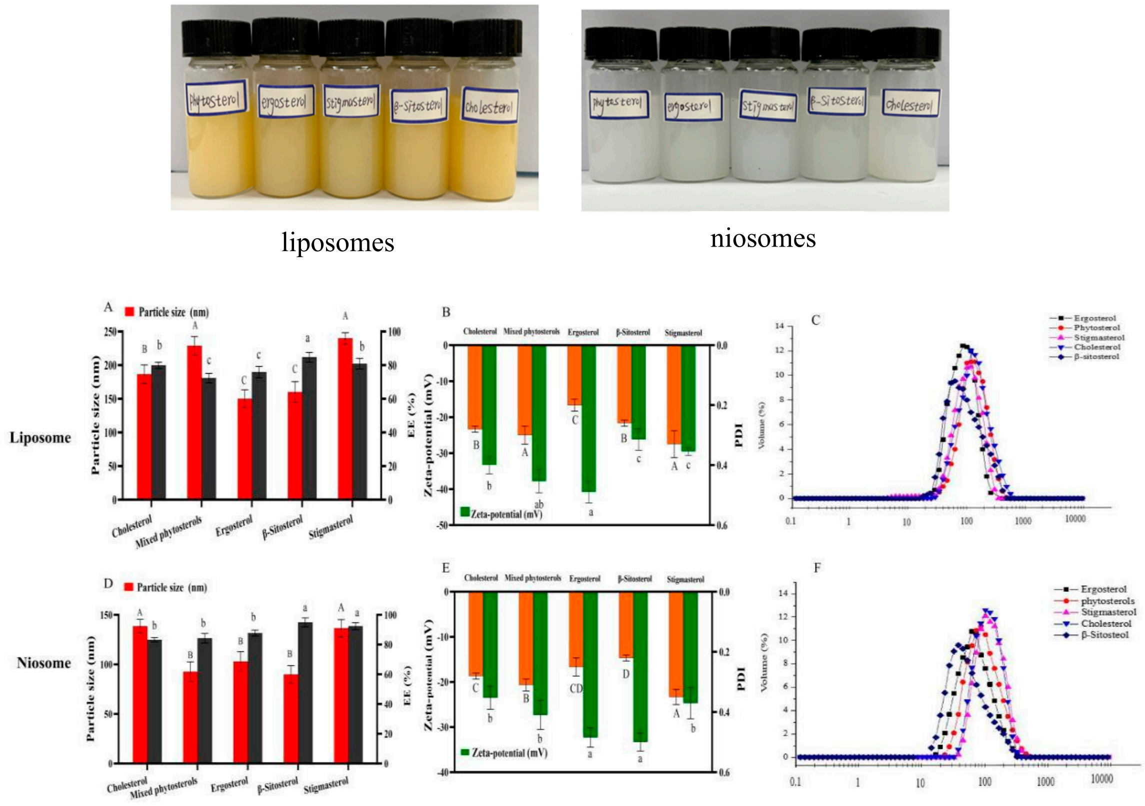

Figure. S1. Visualization (a and b), encapsulation efficiency, particle size (c and f), ζ-potential, PDI (d and g) and particle size distribution (e and h) of GCAPS liposomes and niosomes.

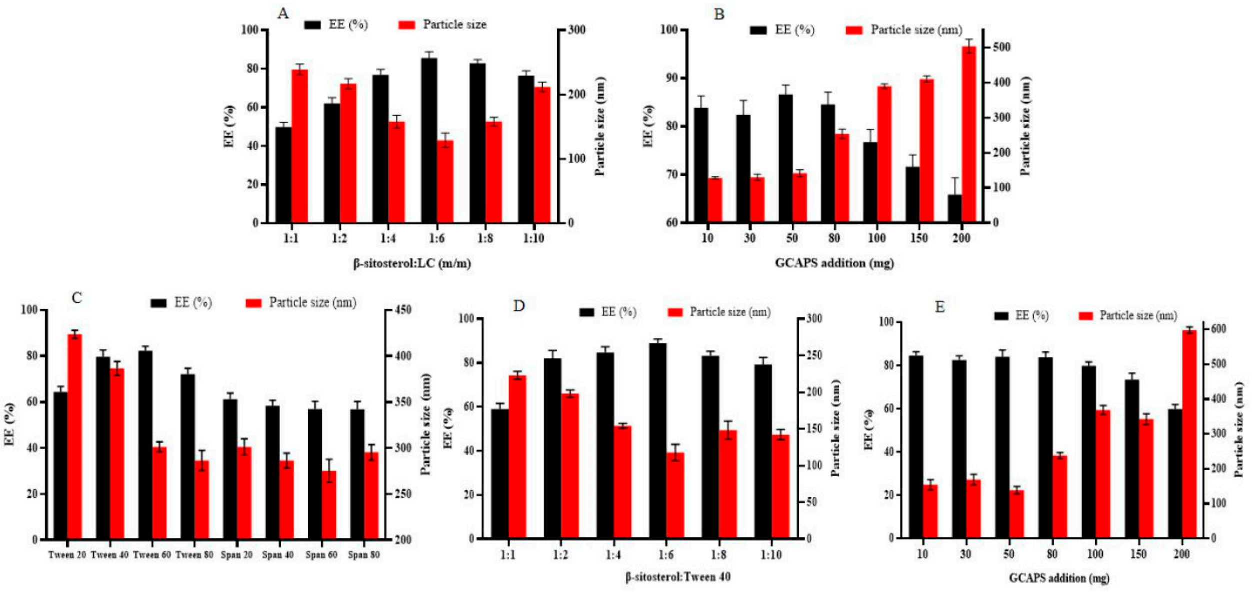

Figure. S2. Single factor experiments for the preparation of liposomes and niosomes loaded with GCAPS (A-E)

The content of phytosterols is critical for the formation and stability of liposomes. Based on the experiments of different phytosterols, β-sitosterol was screened as the best cholesterol substitute. As shown in Fig.1a, When β-sitosterol:LC is 1:1 and 1:2 (m/m), GCAPS leaks due to liposome membrane instability. As the ratio of β-sitosterol increased to 1:6 (m/m), the lipid molecules were more tightly arranged and the packing density increased, which caused the particle size and EE of liposomes to increase. However, excess β-sitosterol can lead to excessive density of the inner space of the lipid bilayer leading to the destruction of the bilayer structure of the liposome, thereby reducing the EE of GCAPS. Therefore, a 1:6 (m/m) ratio of β-sitosterol: LC was used to prepare the liposomes. Fig.1b showed that the effects of GCAPS addition on EE and particle size of liposomes. With the increase of GCAPS content, the load of GCAPS in liposomes initially increased and then decreased.With the increase in GCAPS addition, its loading capacity initially increased and then decreased. When the GCAPS addition was 50 mg, both GCAPS and GWPPG liposomes and niosomes exhibited relatively high encapsulation efficiency and smaller particle sizes.

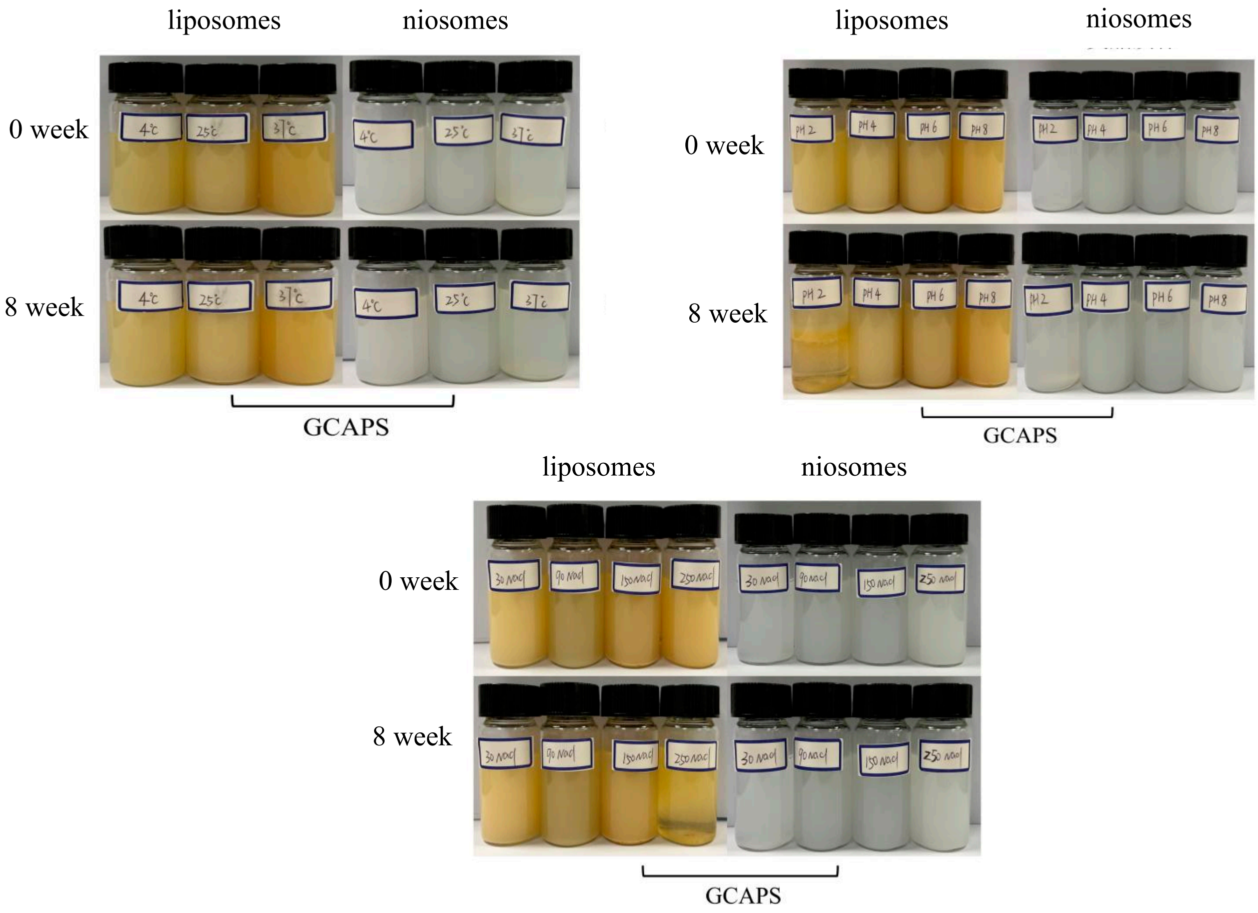

Figure. S3. Visualization of GCAPS encapsulation systems stored at different temperature (4 ℃, 25℃, and 37℃), pH (2,4,6,8) and NaCl concentration (30,90,150,250 mM).

2.2.Isolation and purification of GCAPS

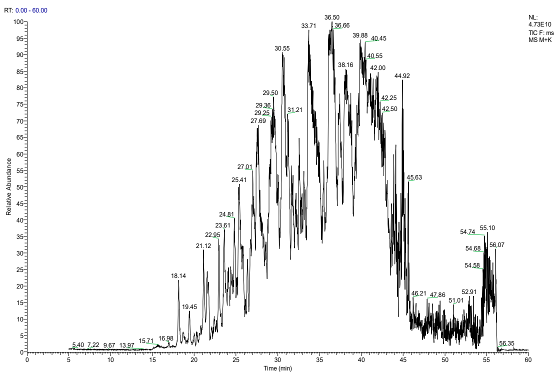

Figure. S4. Total ion chromatograms of GCAP molecular weight <3 kDa ultrafiltration fractions

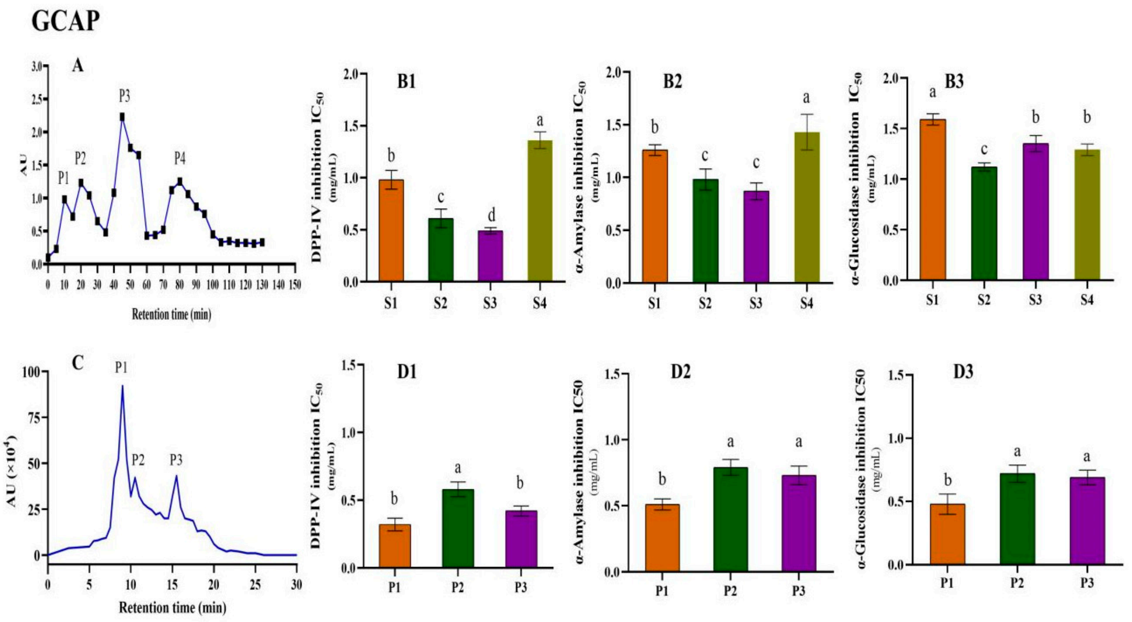

Figure. S5.. Sephadex G-15 gel chromatogram of GCAP and GWPP（A and E）. IC50 values for DPP-IV，α-amylase and α-glucosidase in fractions GCAP-（S1-S4），RP-HPLC profile of partial GCAP-P1（C and G）. IC50 values for DPP-IV，α-amylase and α-glucosidase in fractions GCAP-S3-P1（D1-D3 and H1-H3）. Bars with different letters are significantly different at P < 0.05.

Table S1 Sequence identification, docking energies, and DPP-IV-inhibitory IC50 values of DPP-IV-inhibitory peptides in GCAPS and GWPPG fractions

^{a,b,c,d,e,f}Values followed by different lowercase letters in the same column are significantly different from each other (P < 0.05). Results are expressed as the mean±standard deviation(n=3). ND means not detected.

Sample	Sequence	Length	m/z	RT(min)	DPP-IV inhibition IC50 (μM)	Docking Energy (kcal/mol)	Protein fit
GCAPS	RRHPYF	6	292.4923	17.25	53.44±2.15^{f}	-8.8	A0A452F7Y5
	YPYQ	4	570.2562	18.79	192.34±3.11^{a}	-9.3	P33049
	KFPQY	5	682.3557	22.32	108.65±2.67^{d}	-9.0	P33049
	YPFTQ	5	655.3079	24.35	＞200	-8.5	A0A452ETT8
	WPQYLK	6	417.7282	26.89	85.45±1.68^{b}	-8.5	P33049
	VYPFT	5	626.3172	30.48	＞200	-8.8	A0A452FK23
	GPFPLLLH	8	892.517	34.78	168.34±1.22^{c}	-9.7	A0A452E8B3
	FLPYPYY	7	962.4652	35.99	50.98±1.88^{g}	-9.3	P02670
	VAPFPEVF	8	905.4753	39.47	＞200	-8.7	P18626
	LLIPF	5	602.3899	39.83	62.23±3.44^{e}	-8.9	A0A452FTE6
Control	IPI	3	ND	ND	3.82±0.27^{h}	-9.4	ND

2.3.Determination of intestinal permeability

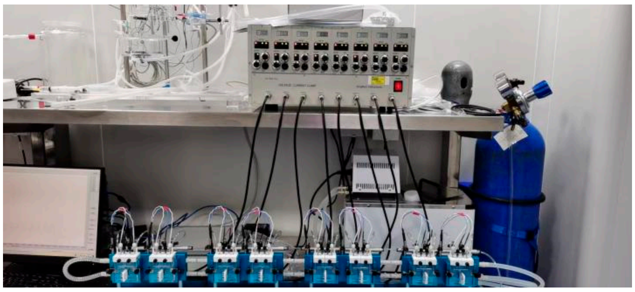

Ussing-chamber equipment

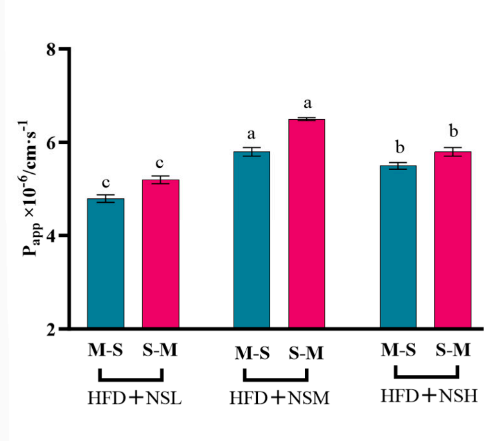

Samples	Er	Particle size
HFD+NSL	0.92±0.02[b]	203.8 ± 7.66 nm
HFD+NSM	0.89±0.01[c]	216.5± 5.44 nm
HFD+NSH	0.95±0.02[a]	286.5± 7.66 nm

Figure. S6. Particle size, apparent permeability parameters, and efflux transport rates of GCAPS-NS at equivalent peptide doses (HFD+NSL, HFD+NSM, and HFD+NSH)

To investigate this phenomenon, we further assessed the intestinal absorption and permeability of liposome formulations at different doses using a Ussing chamber system. Fig.S4 showed that the Papp, Er, and mean particle size of HFD+NSL, HFD+NSM, and HFD+NSH in rat colonic mucosa. Significant differences in Papp among the three groups were observed in both mucosal-to-serosal (M-S) and serosal-to-mucosal (S-M) directions ($P < 0.05$). HFD+NSM exhibited a significantly lower Er compared with HFD+NSL and HFD+NSH. Notably, HFD+NSH displayed an increased particle size relative to HFD+NSM, likely due to higher peptide loading, which promotes a more densely packed internal structure and particle aggregation. Consequently, the larger particle size of HFD+NSH reduced fusion efficiency with the colonic mucosa, leading to a higher Er and lower absorption efficiency. In addition, high peptide loading may alter liposome membrane fluidity or surface properties, further affecting retention and transport across the mucosa.
